# Staphylococcus aureus Bacteremia With Disseminated Multiple Foci and Pyomyositis in an Immunocompetent Patient: A Case Report

**DOI:** 10.7759/cureus.53483

**Published:** 2024-02-03

**Authors:** Ryohei Ono, Izumi Kitagawa

**Affiliations:** 1 General Internal Medicine, Shonan Kamakura General Hospital, Kanagawa, JPN; 2 General Internal Medicine, Shonan Fujisawa Tokushukai Hospital, Kanagawa, JPN

**Keywords:** bacteremia, immunocompetent, disseminated multiple foci, staphylococcus aureus, pyomyositis

## Abstract

Pyomyositis is an uncommon primary infection of skeletal muscle resulting in muscle inflammation followed by pus formation. Pyomyositis is typically caused by Staphylococcus aureus (S. aureus), and most cases are associated with skin penetration and/or immunosuppressive conditions in tropical or even temperate climates. We report a previously healthy, immunocompetent 44-year-old man who presented with fever and right lower back pain. He had received an analgesic injection for his back pain 12 days prior to this visit. His clinical course was further complicated by the coexistence of multiple muscular abscesses, renal infarction, and septic arthritis of the right shoulder. He underwent computed tomography-guided drainage of the abscess. The abscess and blood cultures were positive for methicillin-susceptible *S. aureus*. The patient responded well to prolonged treatment with cefazolin and cephalexin and was discharged 12 weeks after initial admission.

## Introduction

Primary pyomyositis is a rare, acute bacterial infection of the skeletal muscles that results in single or multiple abscesses [1,2]. Pyomyositis is typically caused by Staphylococcus aureus, and staphylococcal pyomyositis is a serious, invasive soft tissue infection that is increasingly recognized even in temperate climates [1-3]. S. aureus bacteremia causes significant morbidity and mortality [4]. The majority of cases of pyomyositis are associated with skin penetration and immunosuppressive conditions; therefore, immunocompetent or healthy cases are not common [1,2]. Treatment choice and duration depend on the diagnosis time, but most cases require antibiotic treatment with surgical or image-guided interventions [5]. We report a case of an immunocompetent patient with S. aureus bacteremia with disseminated multiple foci and pyomyositis.

## Case presentation

A previously healthy, immunocompetent 44-year-old man presented with progressive right lower back pain associated with intermittent fever. The symptoms started 12 days before admission with right lower back pain of abrupt onset without any trauma or exercise. Eleven days prior to admission, he had subsequently consulted an orthopedic surgeon who injected painkillers into his right buttock and prescribed nonsteroidal anti-inflammatory drugs. However, they were ineffective, and he gradually developed intermittent fever. The clinical course was further complicated by arthralgia of the right shoulder and right dorsum of foot pain without any trauma. He was finally transferred to the hospital. He did not have a history of overseas travel, trauma, sexual intercourse with men, or drug abuse.

On arrival, his Glasgow Coma Score was 15, with stable vital signs (blood pressure: 124/73 mmHg; pulse: 83/min; body temperature: 36.8°C; respiratory rate: 20/min). A physical examination showed tenderness, redness, and mild swelling localized on his right upper arm, shoulder, buttock, and dorsum pedis, with a positive sign of psoas. The patient had no peripheral signs, heart murmur, cavities, or scars on the skin. A laboratory investigation on admission showed leukocytosis as follows: white blood cell count, 24,800 cells/μL; 88.1% neutrophils; and C-reactive protein, 37.8 mg/dL. Urinalysis showed pyuria and microscopic hematuria. Glucose and HbA1c levels were within normal limits, suggesting he did not have diabetes mellitus. Human immunodeficiency virus (HIV) antibodies and RNA were also negative.

**Figure 1:**
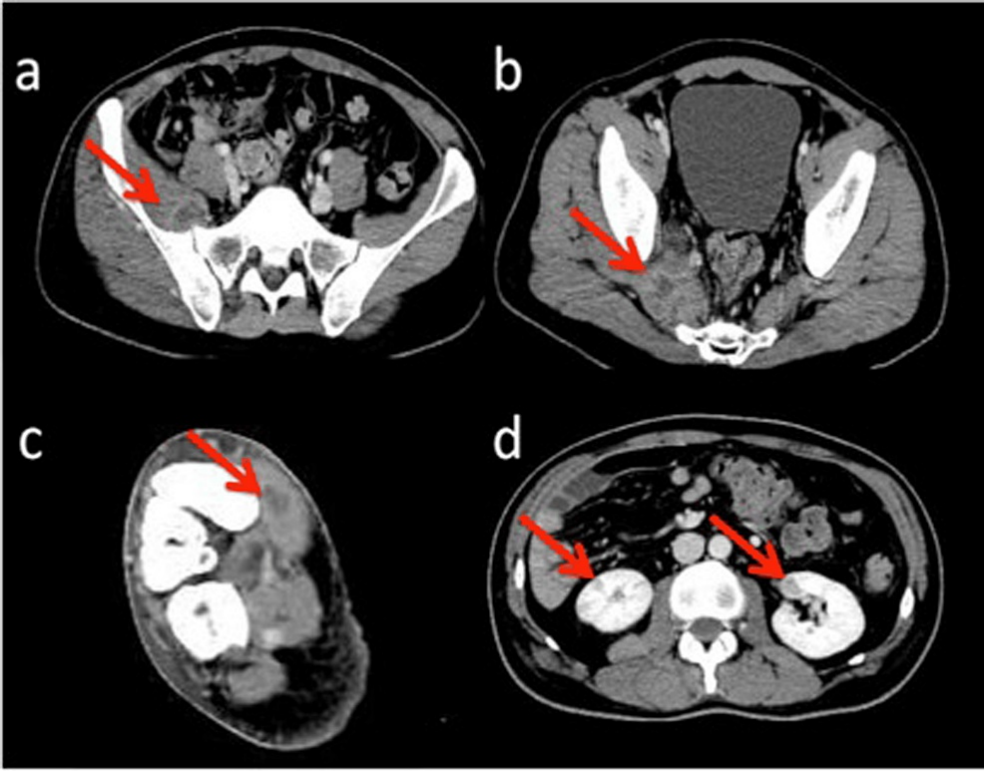
Contrast computed tomography scan at admission. (a: iliopsoas muscle, b: piriformis muscle, c: plantaris muscle, d: renal infarction) Arrows show abscesses.

The patient developed a fever (37.9°C) soon after admission. A contrast CT scan showed abscesses in the iliopsoas, piriformis, and plantaris muscles and renal infarction (Figure 1).

**Figure 2:**
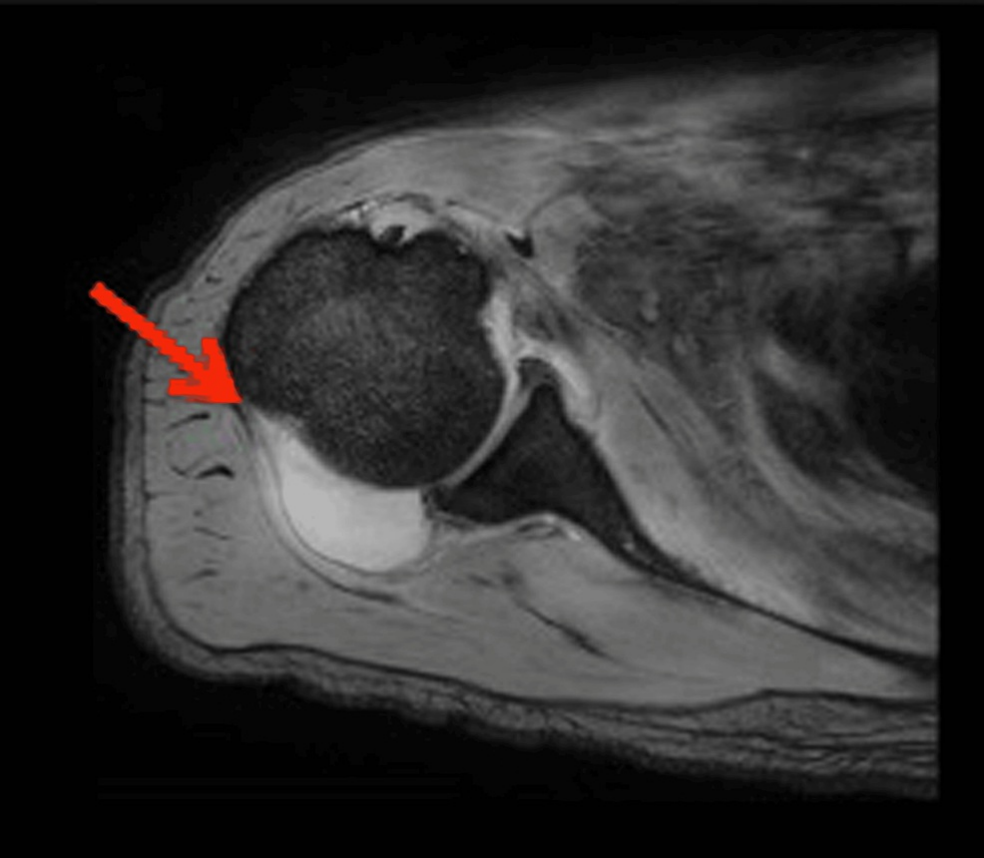
Shoulder T2-weighted magnetic resonance imaging showing arthritis with a high signal.

Ultrasound also showed an abscess in the biceps brachii. Magnetic resonance imaging (MRI) showed septic arthritis of the right shoulder (Figure 2).

**Figure 3:**
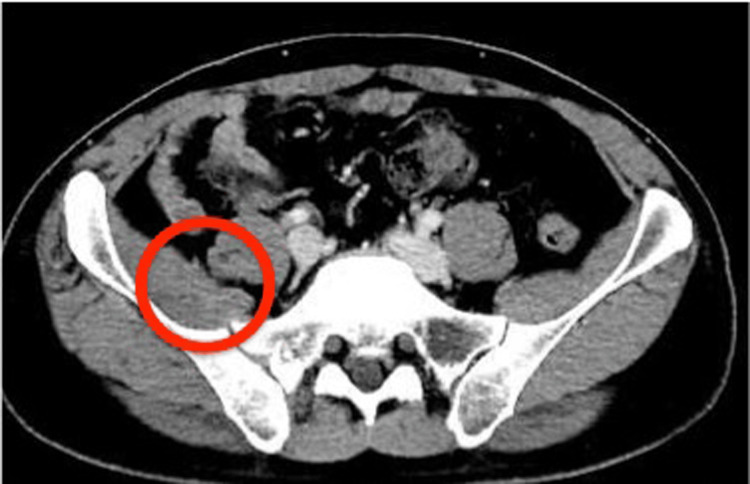
Follow-up contrast computed tomography scan on the 85th day after the admission date showing no abscess in the iliopsoas muscle.

**Figure 4:**
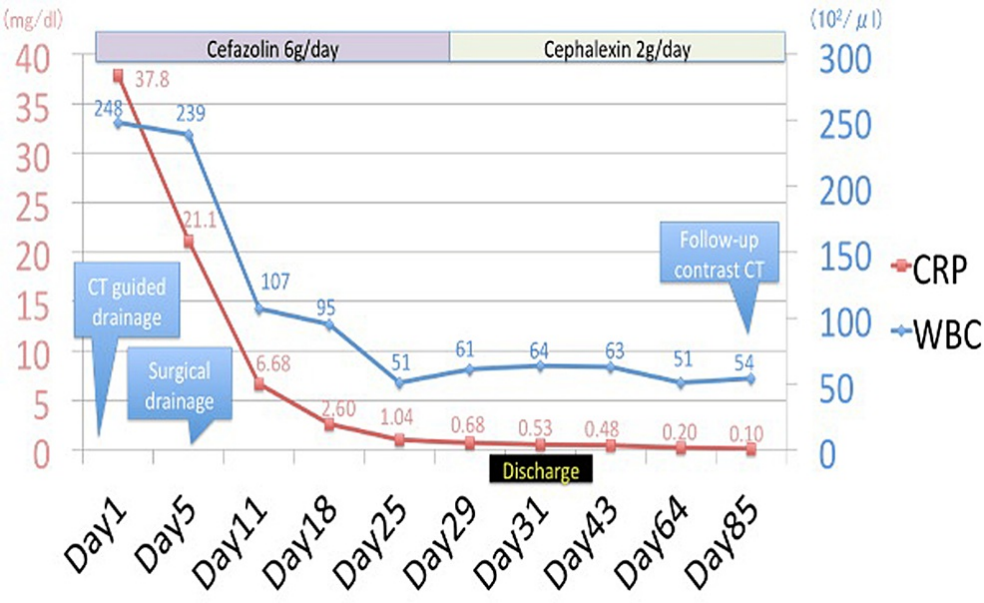
Clinical course.

Initial blood cultures grew methicillin-sensitive S. aureus. Therefore, we diagnosed the patient with S. aureus bacteremia with disseminated multiple foci. He was treated with intravenous cefazolin, 6g per day. Infective endocarditis was also suspected, but the patient denied transesophageal echocardiography. Therefore, we performed transthoracic echocardiography three times, showing no vegetation or regurgitation. One day after admission, the abscess in the piriformis muscle was drained under CT scan guidance. Follow-up blood cultures after 72 hours from initial blood cultures were negative. He also had surgical drainage for the brachii on the fifth day after admission. Gram stain and cultures of the abscess fluid showed S. aureus, which had the same sensitivity as the blood culture. The patient responded well to prolonged cefazolin treatment and drainage for four weeks. He was switched to oral cephalexin 2 g/day, which he had been taking for longer than eight weeks. He was finally discharged after 31 days in the hospital. A follow-up CT scan on the 85th day after admission showed no abscesses (Figure 3). Laboratory data also showed no inflammation (see clinical course in Figure 4).

## Discussion

Pyomyositis, a critical and invasive infection of the soft tissues, carries a significant risk of mortality and is increasingly observed globally, even in regions with milder climates. Pyomyositis is an intriguing disease with unclear pathogenesis. Organisms invade skeletal muscle during transient bacteremia, even though the muscle is inherently resistant to infection in the absence of trauma. Animal studies have shown that intravenous injection of sublethal doses of S. aureus fails to induce infection of the muscle unless the muscles are traumatized by electric shock or ischemia. Normal muscle, if damaged, is susceptible to hematogenous invasion by bacteria with subsequent abscess formation [6,7]. Young males have been reported to be the most susceptible group to this disease. The highest incidence of pyomyositis is between 10 and 40 years of age. Pyomyositis usually affects the large muscles in the buttocks and quadriceps, but the shoulders are rarely affected [8,9].

In most cases of pyomyositis, predisposing factors include skin penetration and immunosuppressive conditions (HIV infection, diabetes mellitus, cancer, connective-tissue diseases, cirrhosis) [1,2]. In our case, the patient was not immunodeficient, diabetic, or HIV-infected. However, we considered our patient developed this complication because of skin penetration of a painkiller injection into his right buttock.

The causative microorganism of pyomyositis is S. aureus in 90% of tropical cases and in up to 70% of temperate cases [10,11]. Another 1-5% of cases involve group A streptococcus and other rare species [3,10]. Methicillin-resistant S. aureus spp. has recently been increasing in frequency. In a study involving S. aureus bacteremic adults in Taiwan, 16.7% (5/30) of community-acquired methicillin-resistant S. aureus bacteremic patients and 20.5% (38/185) of methicillin-susceptible S. aureus bacteremic patients presented with osteomyelitis or septic arthritis [12,13]. S. aureus bacteremia causes significant morbidity and mortality. The overall in-hospital mortality is 28% in immunocompetent patients [4]. The mortality rate of pyomyositis in temperate cases is 6.0-9.4% [10, 14].

Pyomyositis manifests in a progression of three distinct stages, varying in severity. The initial stage, known as the invasive stage, is marked by the muscle becoming progressively swollen and painful due to bacterial infiltration. During this phase, there's no formation of an abscess, and needle aspiration typically shows no significant findings. The condition escalates to the second stage, termed the suppurative phase, within 10 to 21 days. This stage is identified by the development of an abscess. At this point, the majority of pyomyositis cases, approximately 90%, are diagnosed. Biopsy results typically reveal swelling of the muscle fibers, infiltration by lymphocytes, and the onset of suppuration, indicating the replacement of normal muscle tissue with pus. The final stage of pyomyositis is the most severe, characterized by the onset of septicemia, the formation of abscesses in multiple sites, and the dysfunction of multiple organs. This stage is notably linked with a significantly increased risk of mortality [15-17]. Bacteremia, sepsis, acute renal failure, acute respiratory distress syndrome, and metastatic abscesses are some of the complications of pyomyositis [2]. Our patient's presentation is compatible with the final stage because he had bacteremia with disseminated multiple foci, including renal infarction.

Identifying pyomyositis can be challenging due to the lack of distinctive early symptoms; commonly, the initial indications include fever, muscle soreness, and swelling [10,17]. Laboratory data are also not specific for pyomyositis. Leukocytosis and increased CRP levels or the erythrocyte sedimentation rate are often observed, but this is insufficient for differentiation from other infections [11].

The primary approach for treating pyomyositis involves a combination of antibiotic treatment and surgical abscess drainage. In the early stages of the disease, treatment typically involves solely using antibiotics. The chosen antibiotics should be effective against S. aureus, with adjustments made based on results from culture tests and antibiotic sensitivity. Treatment usually starts with a four-week course of intravenous antibiotics, though the duration may be altered depending on the patient's clinical progress and imaging findings. Advanced stages of pyomyositis often necessitate more invasive procedures, including surgical drainage or percutaneous catheter drainage, which should be performed under the guidance of CT scans or ultrasound, in addition to continued antibiotic therapy [14]. It's important to highlight that utilizing CT scans or ultrasound to guide the drainage of abscesses has proven effective, serving diagnostic and therapeutic roles [8].

## Conclusions

Pyomyositis, a condition gaining increasing recognition, poses a serious risk of fatality if not promptly identified and treated effectively. Immediate and proactive diagnosis and management are crucial to averting the risks of sepsis and the spread of metastatic abscesses. Physicians should be vigilant, particularly in patients presenting with fever and muscle pain following any skin penetration procedures, and consider pyomyositis as a differential diagnosis, even in the absence of immunosuppression. This report highlights a case of Staphylococcus aureus bacteremia with multiple disseminated foci and pyomyositis in a patient without immunodeficiency. The successful treatment of our patient, involving extended antibiotic therapy and drainage, underscores the importance of timely intervention in managing this potentially life-threatening condition.
